# Ultrastructure of an oat cell carcinoma of the bronchus producing an antidiuretic hormone.

**DOI:** 10.1038/bjc.1969.11

**Published:** 1969-03

**Authors:** A. G. Whitelaw

## Abstract

**Images:**


### ULTRASTRUCTURE OF AN OAT CELL CARCINOMA OF THE BRONCHUS PRODUCING AN ANTIDIURETIC HORMONE

A. G. L. WHITELAW

*From the Department of Pathology, University of Cambridge, Cambridge* *

Received for publication October 10, 1968

ELECTRON microscope studies of oat cell carcinoma of the bronchus have been carried out by several groups of workers (Laval, 1966; Nagaishi *et al.*, 1965; Obiditsch-Mayer and Breitfellner, 1968; Pariente, Even and Brouet, 1966; Stoebner *et al.*, 1967). No electron microscope paper has yet been published on a bronchial carcinoma known to be secreting antidiuretic hormone. In this paper, the structure of an oat cell carcinoma producing an antidiuretic hormone is described and compared with that of an oat cell carcinoma without endocrine activity. Certain features, not previously reported in oat cell carcinoma, are described.

### MATERIALS AND METHODS

Small pieces of tumour were removed from patients during surgery, immediately placed in buffered osmium tetroxide at 4° C. for 2 hours, dehydrated with ethanol and then embedded in Araldite (Davis, 1959). Sections were cut on a Huxley ultramicrotome, mounted on nitrocellulose-coated copper grids and stained with lead citrate (Reynolds, 1963). Over 600 electron micrographs were taken with a Siemens Elmiskop 1b. Larger blocks of tissue from the same areas were fixed in formol saline, embedded in paraffin wax, sectioned and stained for light microscopy.

### RESULTS

Patient A was diagnosed as carcinoma of the bronchus by radiography, and had no symptoms of endocrine disorder. Histologically tumour A consisted of a random arrangement of small polygonal cells with prominent nuclei and scanty cytoplasm. Mitoses were frequent. The appearance was typical of oat cell carcinoma of the bronchus. Both lymphoid cells and necrosis were observed.

Patient B, in addition to radiographic evidence of a tumour, had symptoms of ectopic secretion of antidiuretic hormone. Plasma was hypotonic, the levels of sodium, potassium and chloride ions all being below normal. Urine was hypertonic and when given a large volume of water to drink, the patient failed to excrete it normally. Adrenal failure was excluded as an explanation because plasma and urine corticoid levels were normal, and renal failure was ruled out by the low plasma urea level.

Patient B's symptoms were those of the Schwartz-Bartter Syndrome (Schwartz *et al.*, 1957). Barraclough, Jones and Lee (1966) demonstrated, by animal assay, an antidiuretic factor in the tumours of patients with this syndrome. When assayed by Dr. J. Lee, of Charing Cross Hospital Medical School, the antidiuretic

* Present address: Mr. Andrew G. L. Whitelaw, St. Mary's Hospital Medical School, London, W.2.

TABLE I.—*Biochemical Findings in Patient B with Oat Cell Carcinoma of the Bronchus*

| | | | |
|---|---|---|---|
| Serum | Na | 120 mg./100 ml. (normal 138–148 mg./100 ml.) | |
| | K | 3·9 mg./100 ml. (normal4·0–5·5 mg./100 ml.) | |
| | Cl | 92 mg./100 ml. (normal 96–108 mg./100 ml.) | |
| | Urea | 13 mg./100 ml. (normal 20–40 mg./100 ml.) | |
| Plasma | Osmolality | 231 mOsm/l. (normal 272–284 mOsm/l.) | |
| Urine | Osmolality (early morning) | | 338 mOsm/l. |
| | Osmolality (after drinking 500 ml. of water) | | 309 mOsm/l. |

Data supplied by Dr. P. Stovin, Papworth Hospital, near Cambridge.

activity (ADA) of tumour B was found to be 40 $\mu$-units/mg. of dry tissue. Normal lung contains less than 1 $\mu$-unit of ADA/mg. of dry tissue (Barraclough *et al.*, 1966). The physiological antidiuretic hormone, arginine vasopressin, has also some oxytocic activity (15% of its ADA). When assayed, the oxytocic activity of tumour B was found to vary from 8 to 20 $\mu$-units/mg. of dry tissue. This is more than one would expect from arginine vasopressin alone, and it is suggested that either a substance similar to arginine vasopressin was produced in the tumour, or both arginine vasopressin and another factor with oxytocic activity were produced in the tumour.

Histological examination revealed an oat cell carcinoma with a high mitotic rate (Fig. 1). Some fibrous tissue and a few lymphoid cells were present, but there was no obvious necrosis. The small polygonal cells had prominent nuclei and a little basophilic cytoplasm which did not stain with Gomori's chrome alum haematoxylin-phloxin method for neurosecretory material.

The ultrastructure of both tumours was identical. At low magnifications, most of the tumour appeared as a packed mass of cells ranging in size from 5·5 $\mu$ to 10 $\mu$, with small intercellular spaces. Between a few of the tumour cells, collagen fibres and fibroblasts could be seen and plasma cells were occasionally seen in direct contact with tumour cells. Blood vessels were very uncommon.

The nucleus normally occupied the majority of the area of a sectioned tumour cell. Chromatin was condensed near the nuclear membrane but dispersed within the nucleus. The nuclear membrane was sometimes invaginated and pores could often be seen (Fig. 2). The nucleoli were usually large, often 2 and occasionally 3 being found within 1 nucleus. Dark, convoluted nucleolonema fibres predominated over paler, finely granular material (pars amorpha). No multinucleate cells were seen. Cells were observed in all stages of mitosis but no mitotic abnormalities were noted.

### EXPLANATION OF PLATES

FIG. 1.—A paraffin section of tumour B. Small cells with prominent nuclei are randomly arranged. A metaphase plate can be seen and some fibrous stroma is visible at the bottom of the plate. × 320.

FIG. 2.—Oat cell carcinoma B. Areas of cytoplasm and nucleus of one tumour cell. A nuclear pore can be seen (NP). Mitochondria, rough endoplasmic reticulum (RER) and rosettes of free ribosomes (R) are prominent in the cytoplasm. × 18,400.

FIG. 3.—Oat cell carcinoma B. A cilium is seen here cut longitudinally (C). × 28,000.

FIG. 4.—Oat cell carcinoma B. An area of tumour where the cells show some epithelial organisation. Two cells are lining a cyst. Microvilli (MV), terminal bars (TB), and tonofilaments (TF) can be seen. × 24,000.

1

3

Whitelaw.

2

Whitelaw.

4

Whitelaw.

The cytoplasm of both tumour A and tumour B was undifferentiated. Small amounts of rough endoplasmic reticulum were present (Fig. 2), and also Golgi apparatus with associated vesicles. The mitochondria were sparse and ranged from being long and thin to being rounded. They sometimes contained many long cristae, but usually contained only a few short cristae. Free ribosomes occurred in large numbers and were often arranged in rosettes (Fig. 2). Centrioles and primary lysozomes were noted in many cells but no granules characteristic of mucin or glycogen were observed. When tumour B was compared with tumour A, there was found to be no significant increase in rough endoplasmic reticulum, free ribosomes, Golgi apparatus or cytoplasmic vesicles.

Cell surfaces were usually smooth, and interdigitations of adjacent cell membranes were very uncommon. A few cilia were observed in both tumours (Fig. 3). Desmosomes were very rare throughout most of the 2 tumours, but occasional regions showed some epithelial arrangement (Fig. 4). A number of cells could be seen lining a cyst, microvilli projecting into its lumen. Where the cell membranes were in contact at the luminal surface, they were joined by terminal bars, with tonofilaments extending into the cytoplasm.

### DISCUSSION

Whenever surgical biopsy material is used for electron microscopy, there is the risk that anoxic changes in the cell structure may occur before the tissue can be immersed in the fixing fluid (e.g. mitochondria and endoplasmic reticulum may swell). Ultrastructural observations are not valid from such tissue. In both cases reported here, the mitochondria and endoplasmic reticulum were minimally swollen and fixation was judged to be adequate.

Cilia have been reported in oat cell carcinoma (Stoebner *et al.*, 1967). Since the bronchial epithelium contains ciliated cells and since we have observed cilia in the intermediate cells of that epithelium, the presence of cilia in tumour cells probably has no more significance than to confirm the origin of the oat cell carcinoma from the intermediate layer of the bronchial epithelium.

Triple nucleoli have not been previously reported in oat cell carcinoma. Nagaishi *et al.* (1965) noted large, fibrous nucleoli as features of lung cancer cells, but the significance of multiple, as distinct from large, nucleoli, remains obscure.

Despite a thorough search, no difference in structure between the 2 tumours, A and B, was found. Tumour B showed no increase relative to A in protein synthesising or secretory structures which one might implicate in the production of an antidiuretic hormone. In the cells which physiologically produce antidiuretic hormone (i.e. the neurones of the supra-optic and para-ventricular nuclei), Green (1966) has recognised 3 types of vacuole: (i) clear vacuoles (diameter 500 Å) resembling synaptic vesicles found throughout the nervous system; (ii) clear vacuoles (diameter 1000 Å); (iii) dense vacuoles ranging in diameter from 100 m$\mu$ to 1200 m$\mu$. It is not yet established in which of these vacuoles the hormone is contained. Although clear vacuoles of diameter 500–1000 Å were observed in tumour B, they appeared with equal frequency in tumour A (Fig. 4). We have also observed them in the intermediate cells of normal human bronchial epithelium.

The lack of any obvious cellular organisation suggesting hormone production in tumour B is not surprising if it is borne in mind that the mass of tumour is many times greater than the mass of the tissue which physiologically secretes the hormone and to produce a comparable amount of hormone, the activity per tumour cell could be very much less than for a normal endocrine cell. Ectopic hormone secretion may be regarded, not as a specialised activity, but as an example of dedifferentiation, possibly the result of a gene, present but normally repressed in the bronchial epithelium, which has become activated in the genetic disruption involved in neoplastic change.

SUMMARY

Using surgical biopsy material, the ultrastructure of an oat cell carcinoma of the bronchus shown to be producing an antidiuretic hormone by assay, was compared with that of an oat cell carcinoma without associated endocrine symptoms. No differences were found and it is suggested that the hormone production is symptomatic of dedifferentiation rather than specialisation in the cancer cell. Cilia, triple nucleoli, and areas of epithelial organisation are all pointed out as unusual features in oat cell carcinoma.

Supported by a research grant from the Durham Fund, King's College, Cambridge.

The author gratefully acknowledges the help of Dr. J. M. G. Davis and Mrs. M. A. Stephens.

### REFERENCES

BARRACLOUGH, M. A., JONES, J. J. AND LEE, J.—(1966) *Clin. Sci.*, **31**, 135.
DAVIS, J. M. G.—(1959) *Nature, Lond.*, **183**, 200.
GREEN, J. D.—(1966) 'The Pituitary Gland', London (Butterworth), p. 240.
LAVAL, P.—(1966) *Revue Tuberc.*, **30**, 844.
NAGAISHI, C., OKADA, Y., DAIDO, S., GENKA, K., IKEDA, S. AND KITANO, M.—(1965) *Expl Med. Surg.*, **23**, 177.
OBIDITSCH-MAYER, I. AND BREITFELLNER, G.—(1968) *Cancer, N.Y.*, **21**, 945.
PARIENTE, R., EVEN, P. AND BROUET, G.—(1966) *Revue Tuberc.*, **30**, 814.
REYNOLDS, E. S.—(1963) *J. Cell Biol.*, **17**, 208.
SCHWARTZ, W. B., BENNETT, W., CURELOP, S. AND BARTTER, F. C.—(1957) *Am. J. Med.*, **23**, 529.
STOEBNER, P., CUSSAC, Y., PORTE, A. AND LE GAL, Y.—(1967) *Cancer, N.Y.*, **20**, 286.


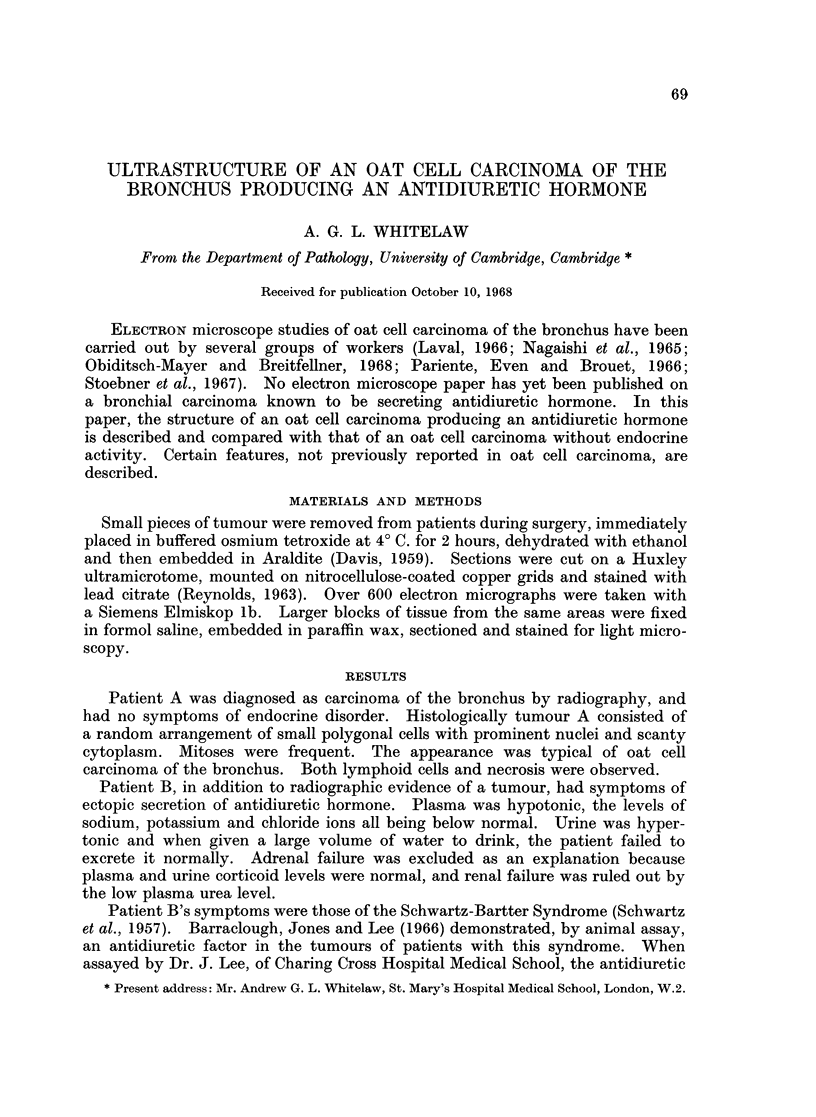

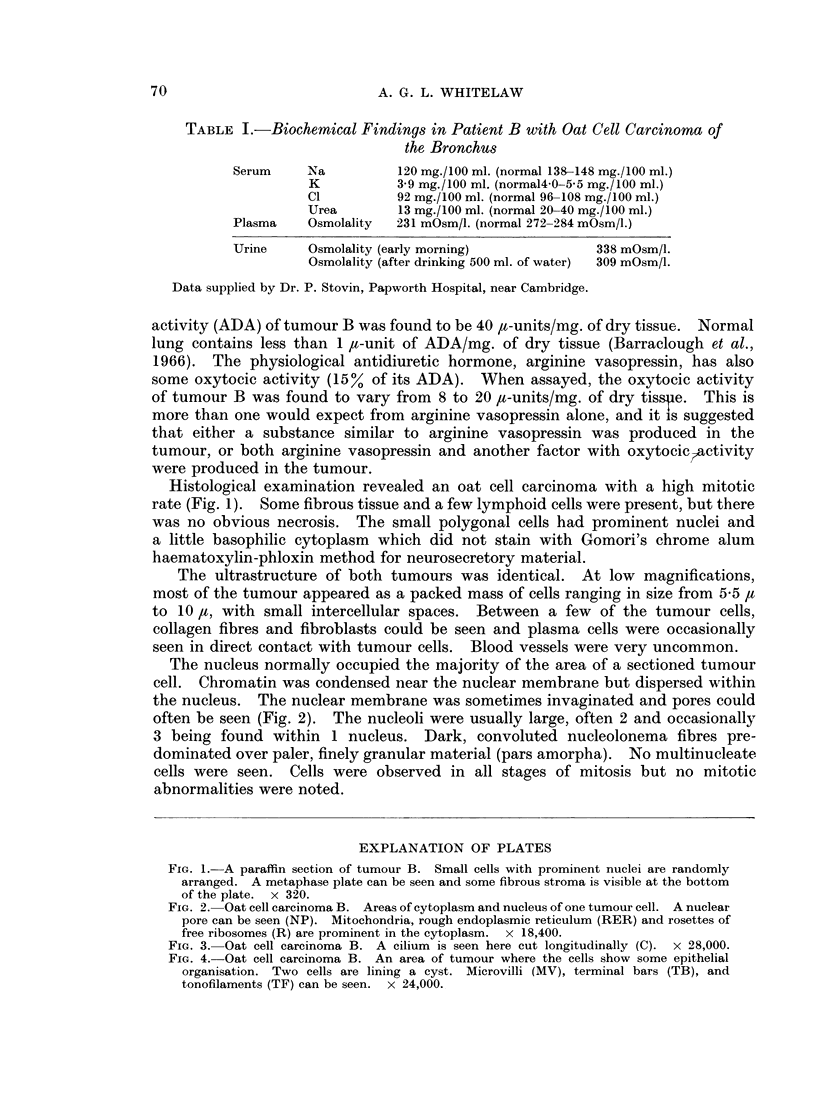

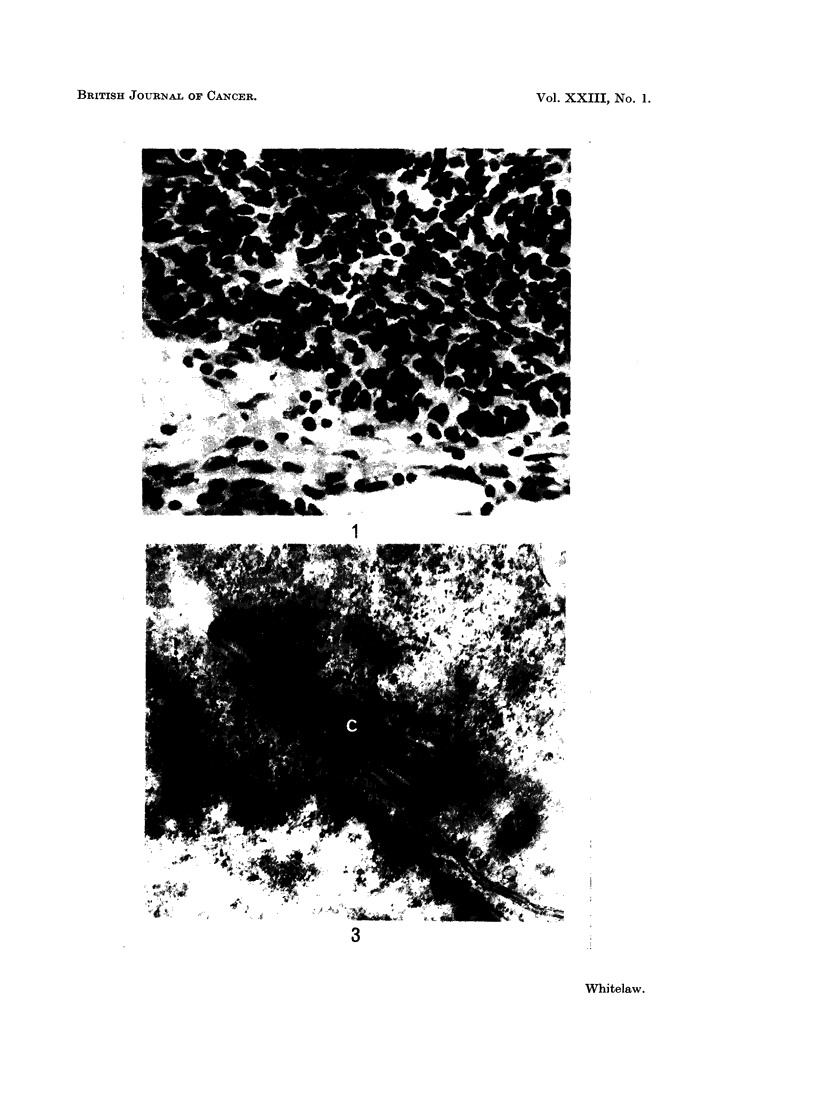

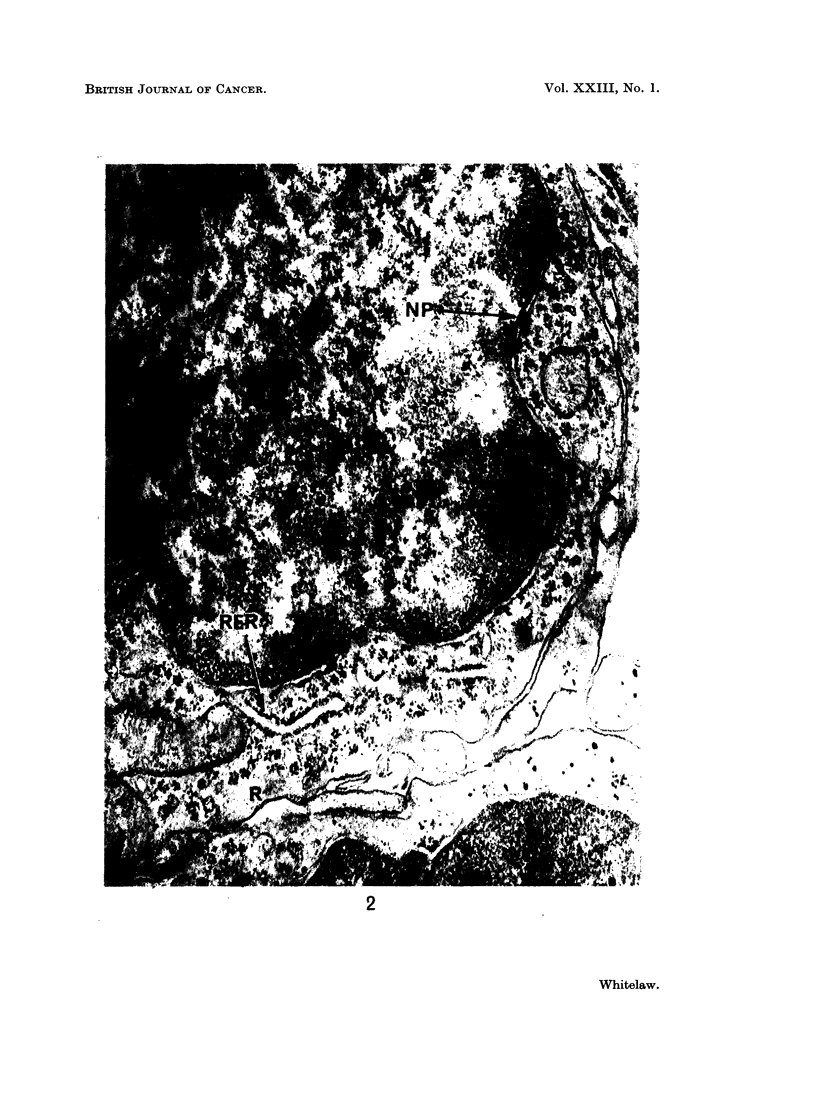

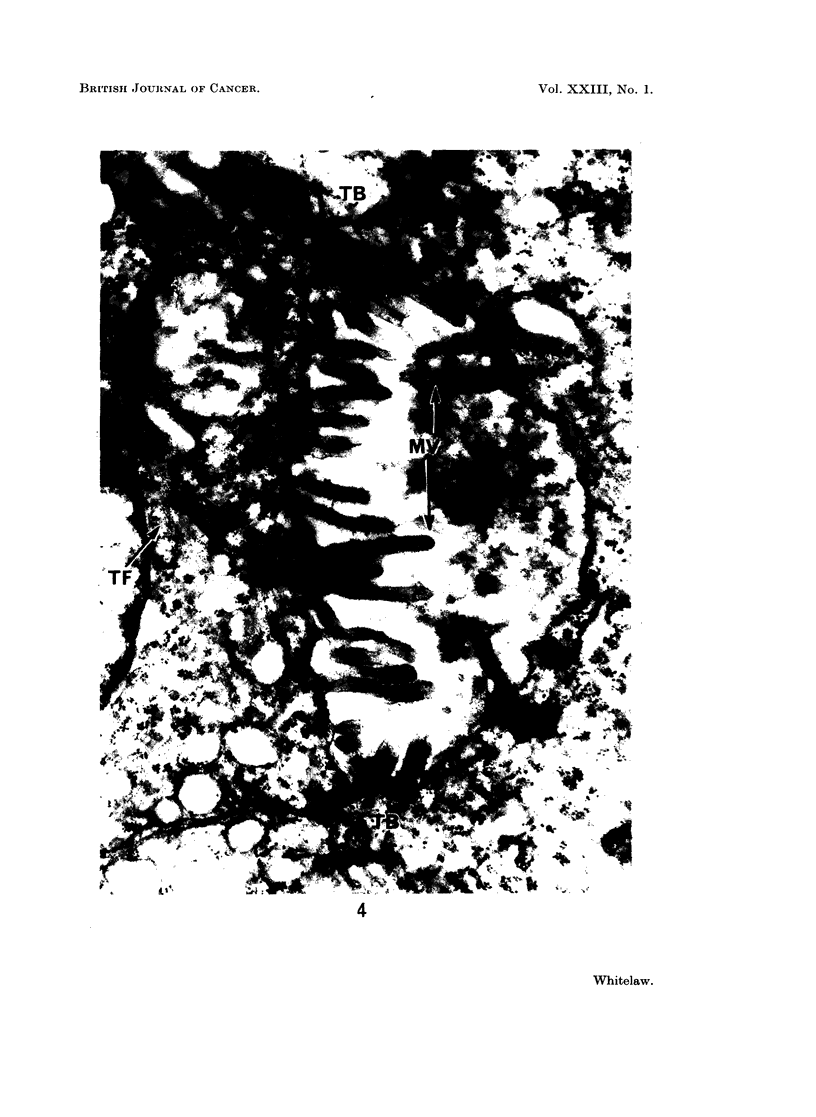

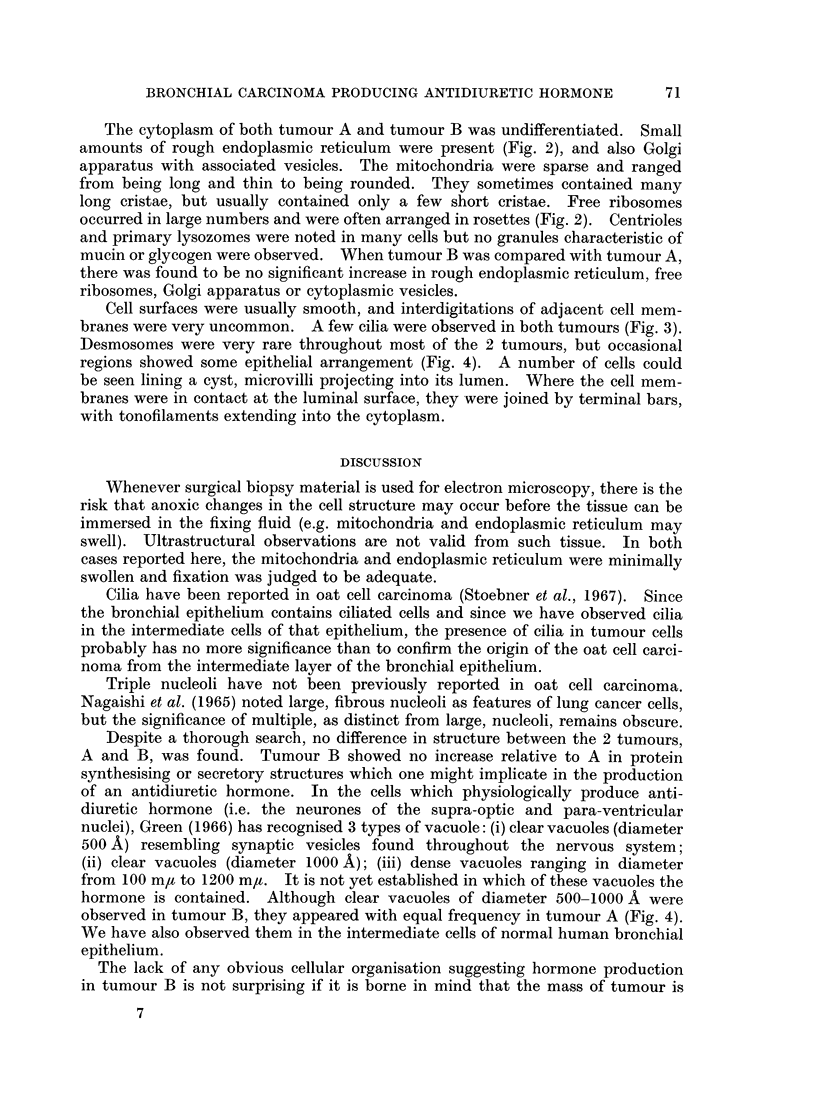

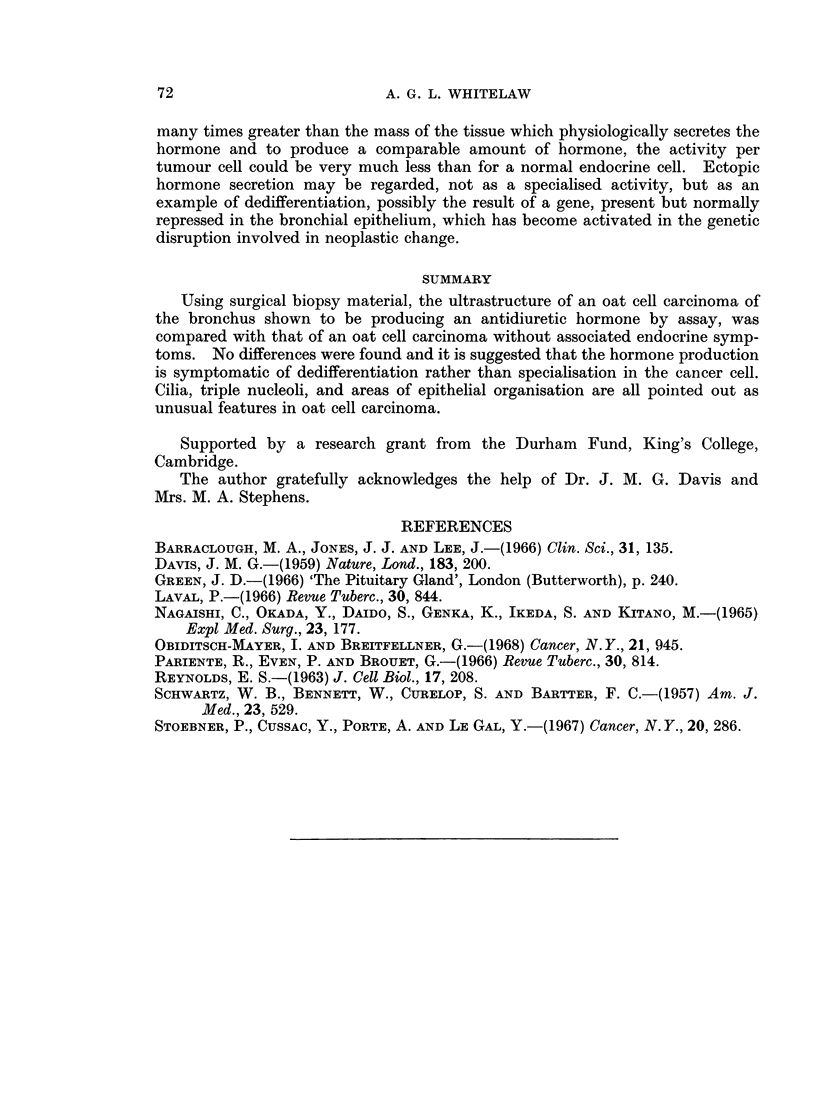


## References

[OCR_00326] Barraclough M. A., Jones J. J., Lee J. (1966). Production of vasopressin by anaplastic oat cell carcinoma of the bronchus.. Clin Sci.

[OCR_00329] DAVIS J. M. (1959). Preparation and sectioning of tissues embedded in araldite for electron microscope examination.. Nature.

[OCR_00330] Laval P. (1966). Cancer anaplasique à petites cellules.. Rev Tuberc Pneumol (Paris).

[OCR_00332] Nagaishi C., Okada Y., Daido S., Genka K., Ikeda S., Kitano M. (1965). Electron microscopic observations of the human lung cancer.. Exp Med Surg.

[OCR_00336] Obiditsch-Mayer I., Breitfellner G. (1968). Electron microscopy in cancer of the lung.. Cancer.

[OCR_00337] Pariente R., Even P., Brouet G. (1966). Ultrastructure des cancers bronchiques à petites cellules.. Rev Tuberc Pneumol (Paris).

[OCR_00340] SCHWARTZ W. B., BENNETT W., CURELOP S., BARTTER F. C. (1957). A syndrome of renal sodium loss and hyponatremia probably resulting from inappropriate secretion of antidiuretic hormone.. Am J Med.

[OCR_00344] Stoebner P., Cussac Y., Porte A., Le Gal Y. (1967). Ultrastructure of anaplastic bronchial carcinomas.. Cancer.

